# Whole genome sequencing of colonies derived from cannabis flowers and the impact of media selection on benchmarking total yeast and mold detection tools

**DOI:** 10.12688/f1000research.53467.1

**Published:** 2021-07-21

**Authors:** Kevin McKernan, Yvonne Helbert, Liam Kane, Nathan Houde, Lei Zhang, Stephen McLaughlin

**Affiliations:** 1 Research and Development, Medicinal Genomics, Beverly, Mass, 01915, USA

**Keywords:** Cannabis, Total Yeast and Mold, Microbiome, Whole Genome Sequencing, qPCR

## Abstract

Background:

Cannabis products are subjected to microbial testing for pathogenic fungi and bacteria. These testing requirements often rely on non-specific colony forming unit (CFU/g) specifications without clarity on which medium, selection or growth times are required. We performed whole genome sequencing to assess the specificity of colony forming units (CFU) derived from three different plating media: Potato Dextrose Agar (PDA), PDA with chloramphenicol and Dichloran Rose Bengal with chloramphenicol (DRBC).

Methods:

Colonies were isolated from each medium type and their whole genomes sequenced to identify the diversity of microbes present on each medium selection. Fungal Internal Transcribed Spacer (ITS3) and Bacterial 16S RNA(16S) quantitative polymerase chain reactions (qPCR) were performed, to correlate these CFUs with fungi- and bacterial- specific qPCR.

Results:

Each plating medium displayed a ten-fold difference in CFU counts. PDA with chloramphenicol showed the highest diversity and the highest concordance with whole genome sequencing. According to ITS3 and 16S qPCR confirmed with whole genome sequencing, DRBC under counted yeast and mold while PDA without chloramphenicol over counted CFUs due to bacterial growth without selection.

Conclusions:

Colony Forming Unit regulations lack specificity. Each medium produces significant differences in CFU counts. These are further dependent on subjective interpretation, failure to culture most microbes, and poor selection between bacteria and fungi. Given the most pathogenic microbes found on cannabis are endophytes which culture fails to detect, molecular methods offer a solution to this long-standing quantification problem in the cannabis testing field.

## Introduction

Total yeast and mold testing are required in many states to test the safety of cannabis, prior to the sale of cannabis flowers and cannabis-infused products. Cannabis is an inhaled product, and cases of cannabis-transmitted *Aspergillosis* have been reported in the clinical literature ( Bal *et al.*, 2010; Gargani *et al.*, 2011; McKernan *et al.*, 2015, 2016; Remington *et al.*, 2015; Ruchlemer *et al.*, 2015). Cannabis is a unique matrix, in that antibiotic cannabinoids can make up to 20% of the flowers’ weight, and many fungi infecting cannabis are endophytes. Endophytes are not easily cultured from the plant without lysing open plant cell walls. The conditions which lyse open plant cells walls also lyse open fungal cell walls, thus impacting the viability of the microbes in the lysis and homogenization processes required for testing. Cannabis flowers contain both bacteria and fungi, further complicating fungal quantification for colony forming units (CFU) that lack speciation. Antibiotic selections are often utilized to reduce background bacteria, but many of these antibiotics (e.g. chloramphenicol) inhibit the growth of the most pathogenic fungi found on cannabis ( *Fusarium*, *Pythium* and *Aspergillus*) ( Smith & Marchant, 1968; Day *et al.*, 2009; Joseph *et al.*, 2015).

As part of an AOAC Emergency response validation (ERV) in the State of Michigan, we investigated the impact of medium selection on surveying total yeast and mold on cannabis. Cured cannabis flowers were homogenized and tested on 3 different plating media. These data were compared to ITS3- and 16S-based qPCR and whole genome sequencing. To further complement these cannabis flower samples, organisms were acquired from the American Tissue Culture Collection (ATCC) and plated as pure monocultures on different plating media to confirm the differential growth on each medium.

## Methods

### Plating

Samples originated from Steadfast Analytical Laboratories (Hazel Park, MI) and were tested independently at a laboratory within the Michigan Coalition of Independent Cannabis Testing Laboratories. Briefly, 10 grams of dried cannabis flowers were sampled from three lots of homogenized cannabis containing high, medium and low quantities of fungal and bacterial CFUs, as measured using culture-based techniques with chloramphenicol selection. 10 grams of homogenized flower were soaked with 90 ml of Tryptic Soy Broth (TSB, Medicinal Genomics #420205) in a filtered Nasco Whirl-Pak bag (#B01385). Samples were homogenized by hand, and then 0.1 mL of solution plated onto three media (DRBC, PDA with chloramphenicol, PDA, at 1:100 dilution). Two additional dilutions were prepared (10 mL into 90 mL) and the same plating protocol was followed. All plates were incubated for 5 days at 25°C.

### qPCR

ITS3 qPCR was performed as described in McKernan *et al.* with two modifications. Briefly, 1ml of homogenate from a Whirl-Pak bag was collected and briefly micro-centrifuged to enrich for live organisms. This pellet was resuspended in 200 μl ddH _2_O and lysed with the addition of 12 μl of Thaumatin-like protein (TLP) and incubated at 37°C for 30 minutes. This enzymatic lysis step (glucanase) ensures more complete lysis of fungal cell walls (Medicinal Genomics part #420206, McKernan *et al.*, 2015, 2016). 12.5 μl of MGC Lysis buffer was added, vortexed and incubated for 5 minutes at 25°C. Lysed samples were micro-centrifuged and 200 μl of supernatant was aspirated and added to 250 μl of Medicinal Genomics binding buffer (MGC part# 420001) for magnetic bead isolation. The samples were incubated with the Medicinal Genomics magnetic bead mixture for 10 minutes, magnetically separated and washed two times with 70% ethanol. The beads were dried at 37°C for 5 minutes to remove excess ethanol and eluted with 25 μl of ddH _2_O. Quantitative PCR was performed using Medicinal Genomics PathoSEEK Total Yeast and Mold detection assay (MGC# 420103) and Medicinal Genomics PathoSEEK Total Aerobic Count Assay (MGC# 420106) according to the manufacturers’ instructions on a BioRad CFX96 thermocycler.

### DNA isolation from colonies for whole genome sequencing

A total of 45 colonies were picked with a pipette tip and introduced into 200 μl of ddH _2_O with 12.5 μl of MGC TLP (MGC part #420206). TLP is a glucanase active at 37°C. Samples were digested for 30 minutes at 37°C and 12.5 μl of MGC Lysis buffer was added, vortexed and incubated for 5 minutes at 25°C. Lysed sample were micro-centrifuged and 200 μl of supernatant was aspirated and added to 250 μl of MGC binding buffer (MGC part # 420001) for magnetic bead isolation. The samples were incubated with the bead mixture for 10 minutes, magnetically separated and washed 2 times with 70% ethanol. The beads were dried at 37°C for 5 minutes to remove excess ethanol and eluted with 25 μl of ddH _2_O.

### Library construction for whole genome sequencing.

*Fragmentation*

Genomic DNA (gDNA) was quantified with a Qubit (Thermo Fisher Scientific) and normalized to reflect 4-8 ng/μl in 13 μl of TE buffer. Libraries were generated using enzymatic fragmentation with the NEB Ultra II kits (NEB part # E7103). Briefly, 3.5 μl of 5X NEB fragmentation buffer and 1 μl of Ultra II fragmentation enzyme mix are added to 13 μl of DNA. This reaction was tip-mixed 10 times, vortexed, and quickly centrifuged. Fragmentation was performed in a BioRad CFX96 thermocycler at 3.5 minutes at 37°C, 30 minutes at 65°C. The reaction was kept on ice until ready for adaptor ligation.

*Adaptor ligation*

**Table:** 

Component	Volume (μl)
SureSelect Adaptor Oligo Mix (brown cap)	0.75
ddH _2_O	0.5
Ultra II Ligation Master Mix	15
Ligation enhancer	0.5
**Total Volume**	**16.75**

The master mix for ligation was prepared on ice using 0.75 μl of Agilent SureSelect Adaptor Oligo Mix, 0.5 μl of ddH _2_O, 15 μl of NEB Ultra II Ligation Master Mix, 0.5 μl of Ligation enchancer (New England Biolabs) for a total reaction volume of 16.75 μl.

Ligation was performed by the addition of 16.75 μl of ligation master mix to the 17.5 μl Fragmentation/End Prep DNA reaction mixture, incubate for 15 minutes at 20°C. To purify excess adaptors and adaptor dimers, AMPure XP beads (Beckman Coulter #A63881) were vortexed at room temperature for resuspension and16 μl (approximately 0.45X) of resuspended AMPure XP beads were added to the ligation reactions. This was well-mixed by pipetting up and down at least 10 times. The mixture was incubated for 5 minutes at 25°C. The PCR plate was placed on an appropriate magnetic stand (Medicinal Genomics #420202) to separate the beads from the supernatant. After the solution was clear (about 5 minutes), the supernatant was carefully removed and discarded. We were careful not to disturb the beads containing target DNAmolecules. The magnetic beads were washed by adding 200 μl of 70% ethanol to the PCR plate while on the magnetic stand. Followed incubation at room temperature for 30 seconds, and then careful removal and discarding of the supernatant. The ethanol wash was repeated once for a total of 2 washes. Trace amounts of ethanol were removed. The beads were air dried for ~ 7 minutes while the PCR plate was on the magnetic stand with the lid open. The PCR plate was then removed from the magnet and target DNA eluted from the beads into 10 μl of H _2_O, then 9 μl of cleaned DNA was transferred to a fresh well.

*PCR amplification*

A volume of 12.5 μl 2x NEBNext Q5 Hot Start Master Mix (New England Biolabs #M0492S) was added to 9 μl ligated DNA, then 3.5 μl of NEB 8bp index primer/universal primer were added to the mix. The reaction ran in a cycling program set at 98°C for 30 seconds as an initial denaturization step; six cycles of denaturation, annealing and extension were performed, cycling between 98°C for 10 seconds and 65°C for 75 seconds. A final 5-minute step at 65°C was performed, with a final 4°C forever step.

**Table:** 

Step	Temp	Time	Cycle
Initial denaturation	98°C	30 sec	1
Denaturation Annealing/Extension	98°C 65°C	10 sec 75 sec	6
Final extension	65°C	5 min	1
Hold	4°C	forever	1

*PCR reaction cleanup*

AMPure XP beads were resuspended at room temperature with a brief vortex. A volume of 15 μl of resuspended AMPure XP beads was added to the PCR reactions (~ 25 μl). To mix well, we pipetted up and down at least 10 times. The mixture was incubated for 5 minutes at room temperature. The PCR plate was put on an appropriate magnetic stand to separate the beads from the supernatant. After the solution was clear (about 5 minutes), the supernatant was carefully removed and discarded. We were careful not to disturb the beads containing the target DNA. A volume of 200 μl of 70% ethanol was added to the PCR plate while on the magnetic stand. The mix was incubatedat room temperature for 30 seconds, and then the supernatant was carefully removed and discarded. The ethanol wash was repeated once more. The beads were air dried fof 7 minutes while the PCR plate was on the magnetic stand with the lid open. The target DNA molecules were eluted from the beads into 15 μl of nuclease-free H _2_O, and 15 μl were transferred into a fresh well.

*Sample quality control*

Libraries were evaluated on an Agilent Tape Station prior to pooling for Illumina sequencing. Sequencing was performed by GeneWiz, Cambridge MA. A total of 473 million paired reads (2 × 150bp) were generated, averaging over 10 million read pairs per sample and a total sequence of 141Gb.

## Analysis

Fastq files were uploaded to OneCodex) for Kmer analysis and Simpson’s diversity index analysis for each genome ( *Extended data:* Supplementary Table 1, sheet Summary https://doi.org/10.5281/zenodo.4759883). Reads were also assembled with MegaHit v.1.2.9 ( Li *et al.*, 2015, 2016). The Nextflow mapping and assembly pipeline is published on GitHub. Quast 5.0 was used to calculate the assembly quality statistics ( Gurevich *et al.*, 2013). Sequencing data is deposited in NCBI under Project ID PRJNA725256.

## Results

**Figure 1.**
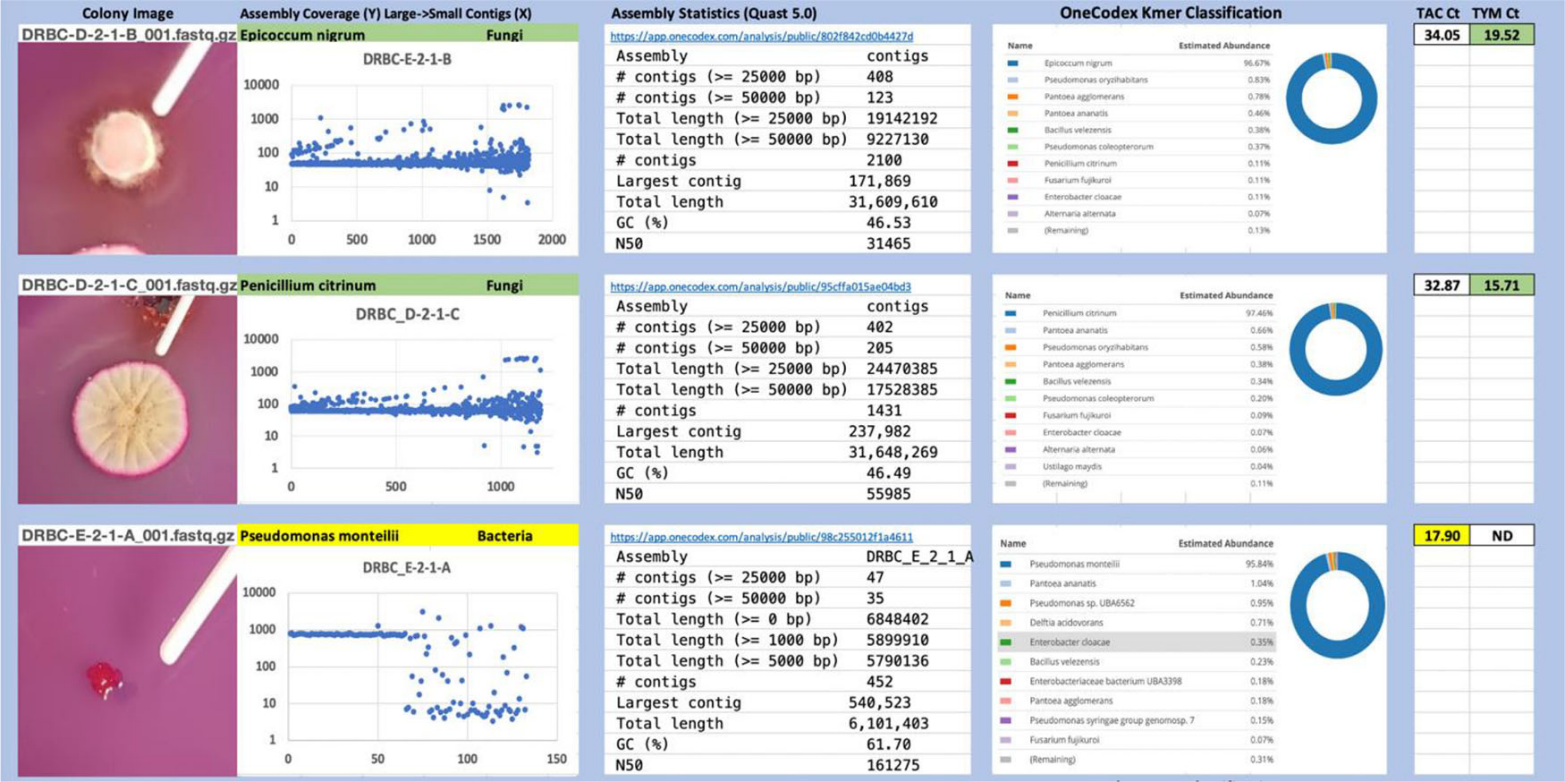

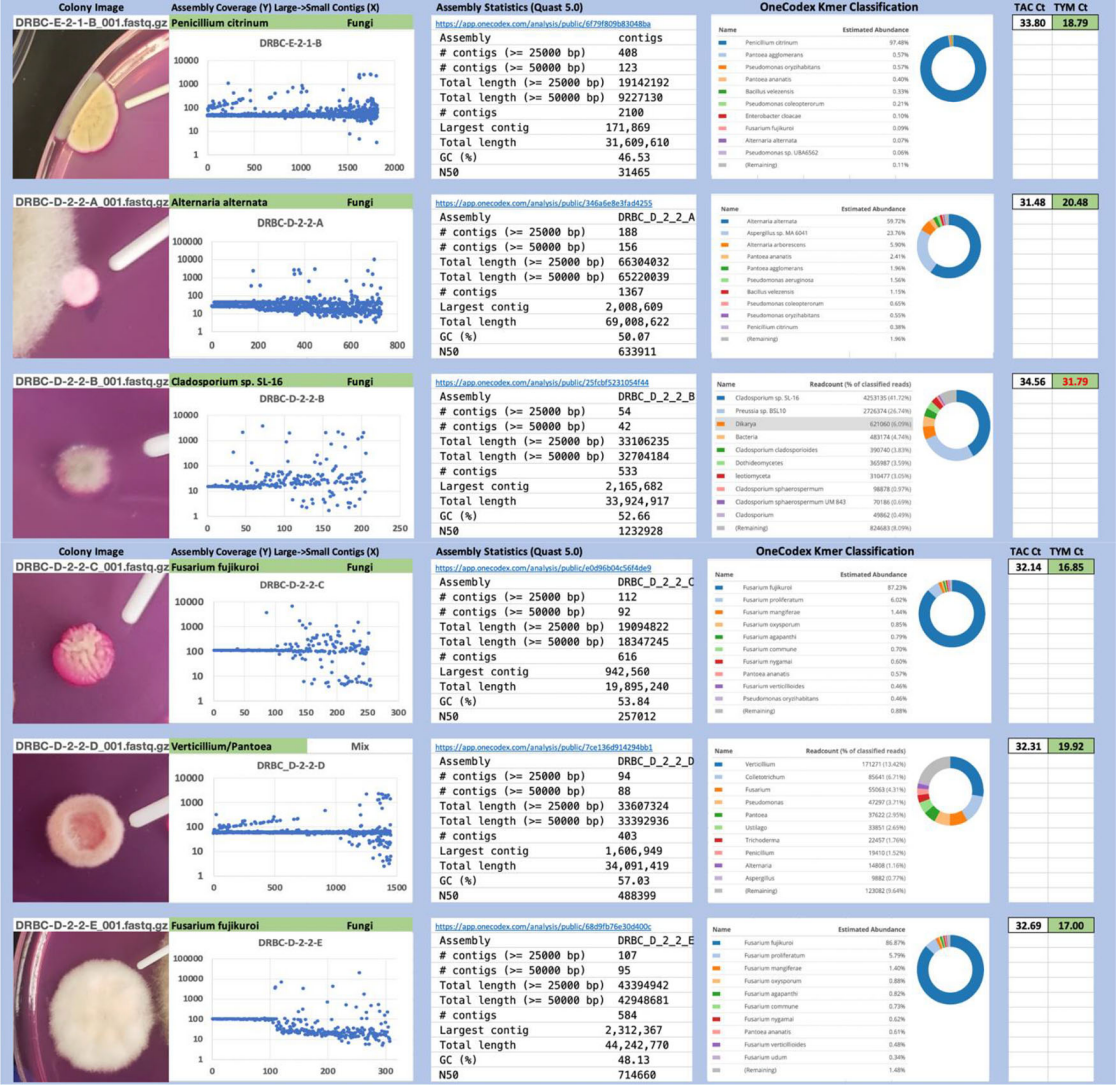

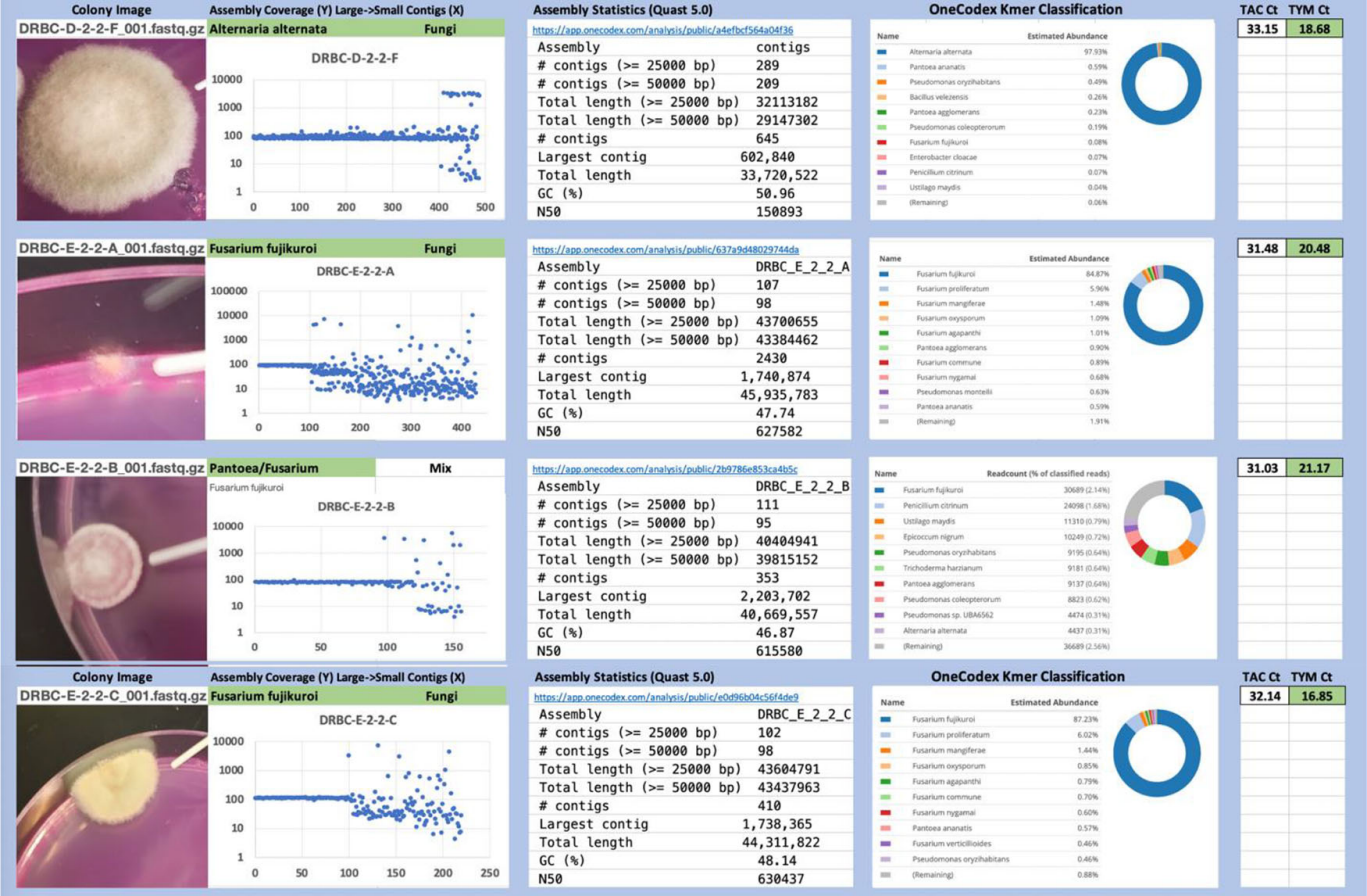
DRBC with chloramphenicol. Colony Image (Left), Assembly sequence coverage (Y) compared with contig Length (X) where the contigs are sorted largest to smallest from left to right (Mid-Left). Assembly statistics calculated with Quast 5.0 (Mid Right). OneCodex speciated Kmer count (Right).

**Figure 2.**
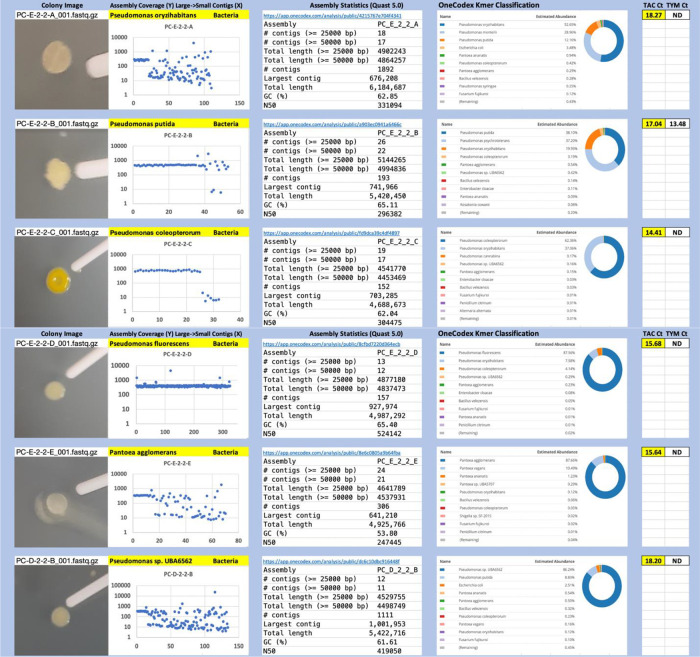

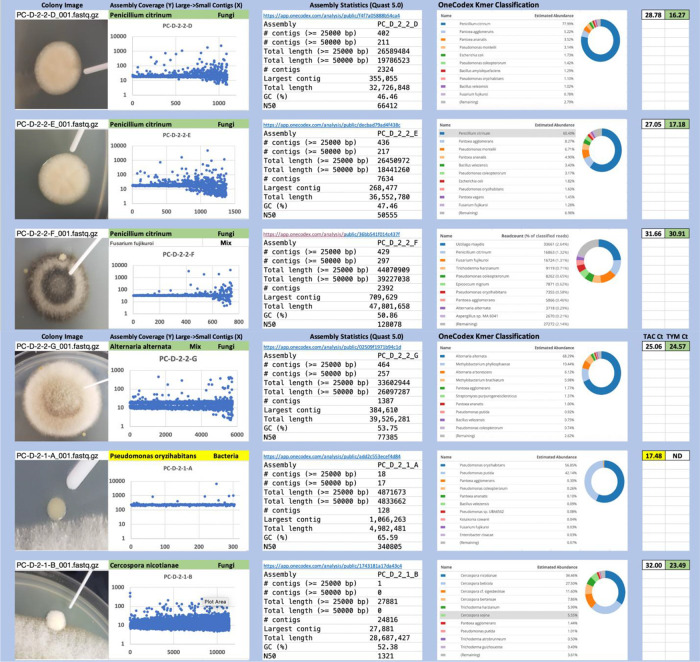

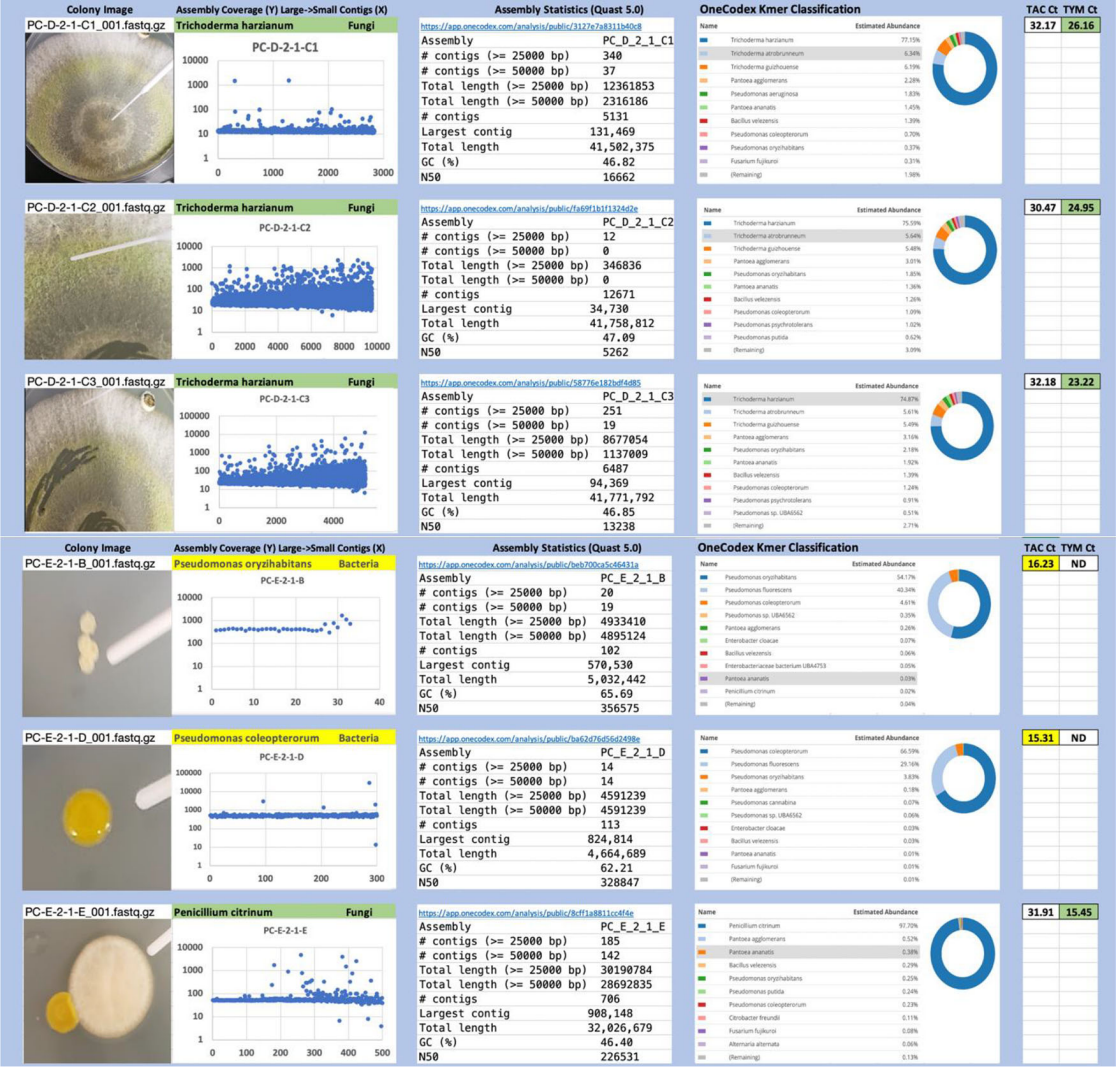
PDA with chloramphenicol. Colony Image (Left), Assembly sequence coverage (Y) compared with contig Length (X) where the contigs are sorted largest to smallest from left to right (Mid-Left). Assembly statistics calculated with Quast 5.0 (Mid Right). OneCodex speciated Kmer count (Right).

**Figure 3.**
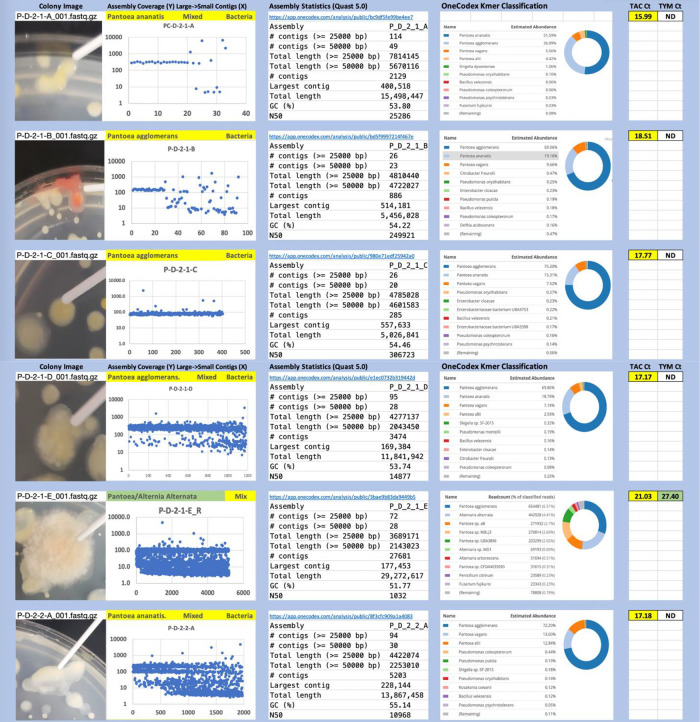

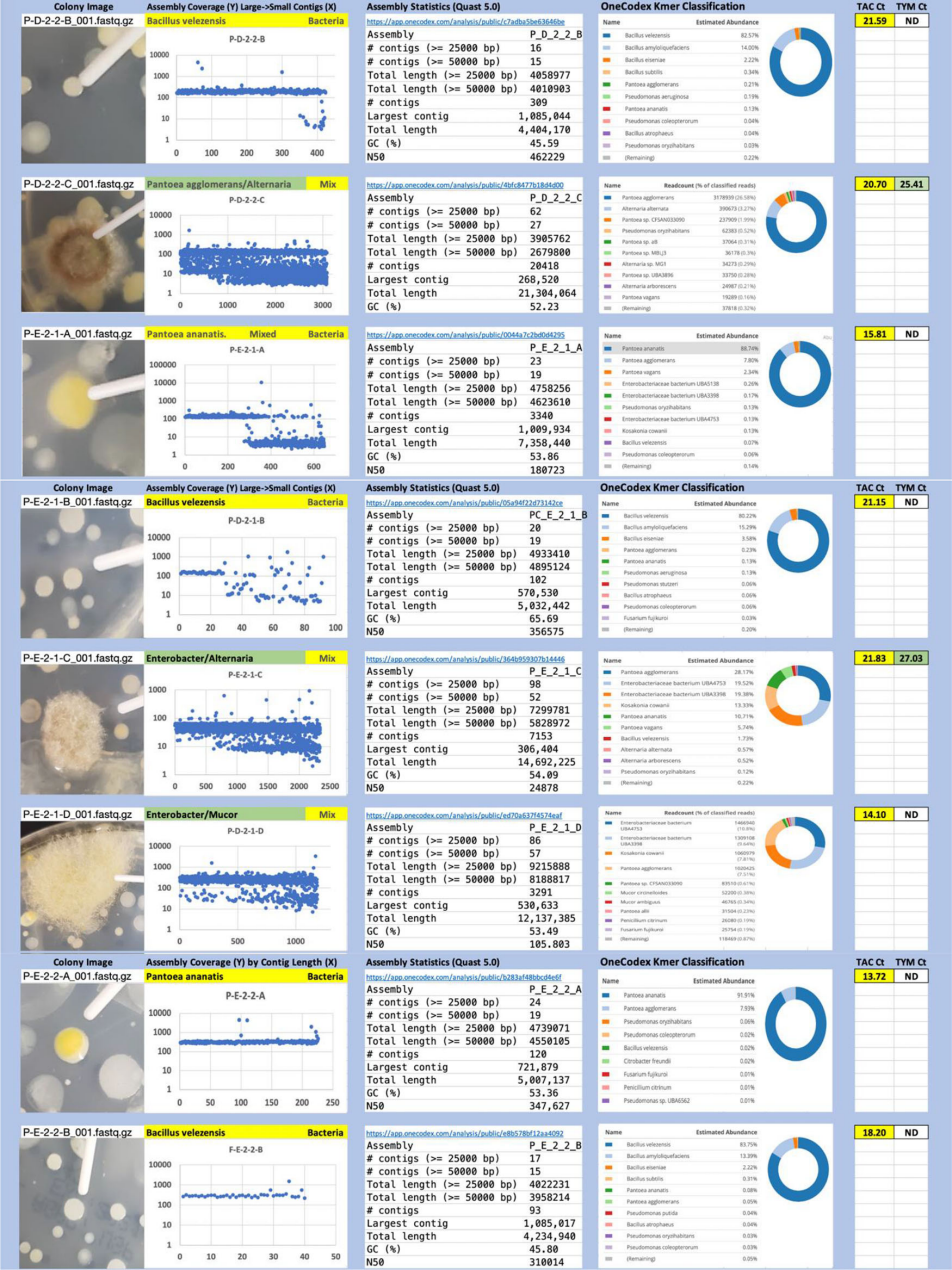
PDA without chloramphenicol. Colony Image (Left), Assembly sequence coverage (Y) compared with contig Length (X) where the contigs are sorted largest to smallest from left to right (Mid-Left). Assembly statistics calculated with Quast 5.0 (Mid Right). OneCodex speciated Kmer count (Right).

**Figure 4.**
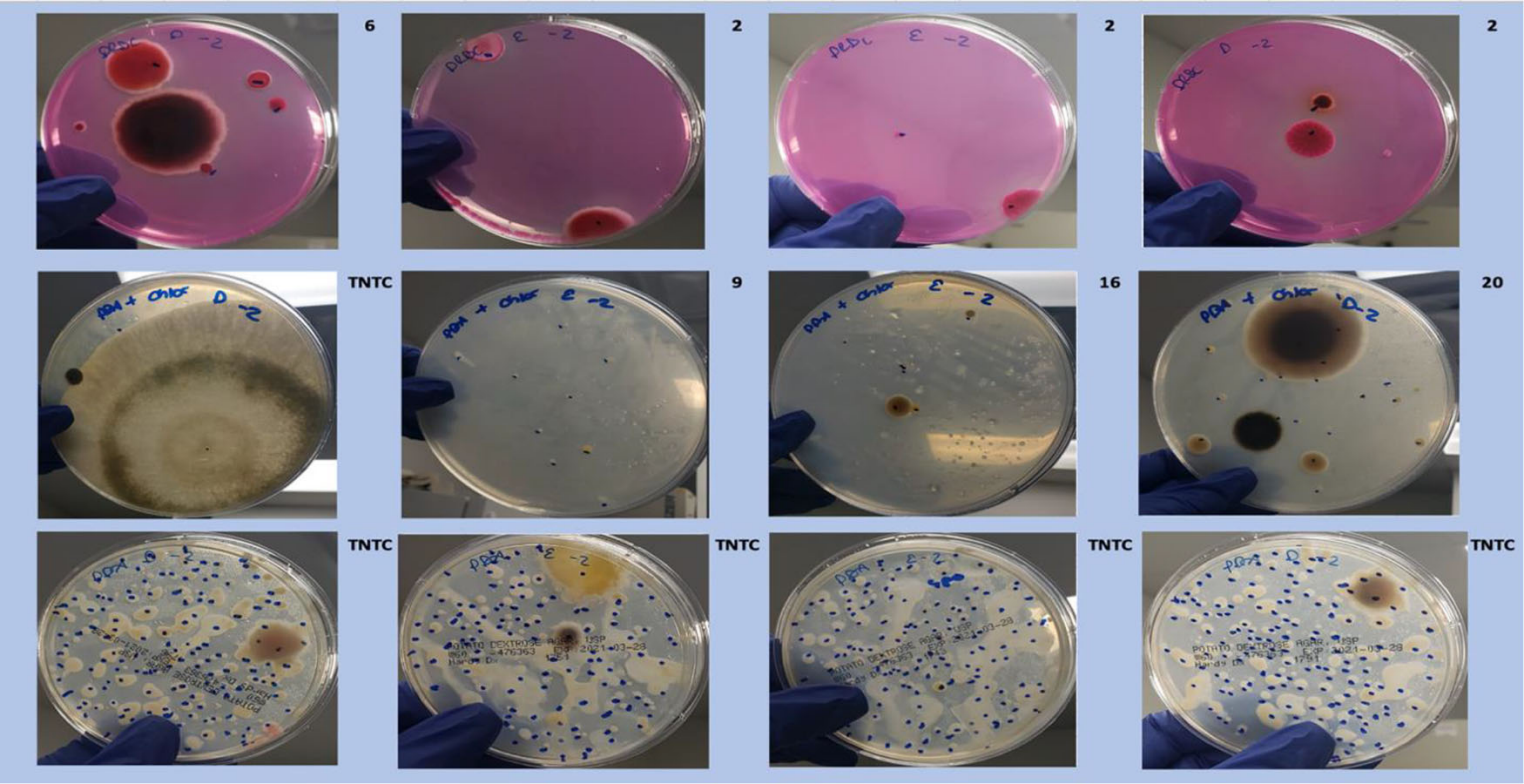
Plating images. Dichloran Rose Bengal (Top). Potato Dextrose Agar with chloramphenicol (PDA – CAMP, Middle). PDA without CAMP (Bottom).

**Figure 5.**
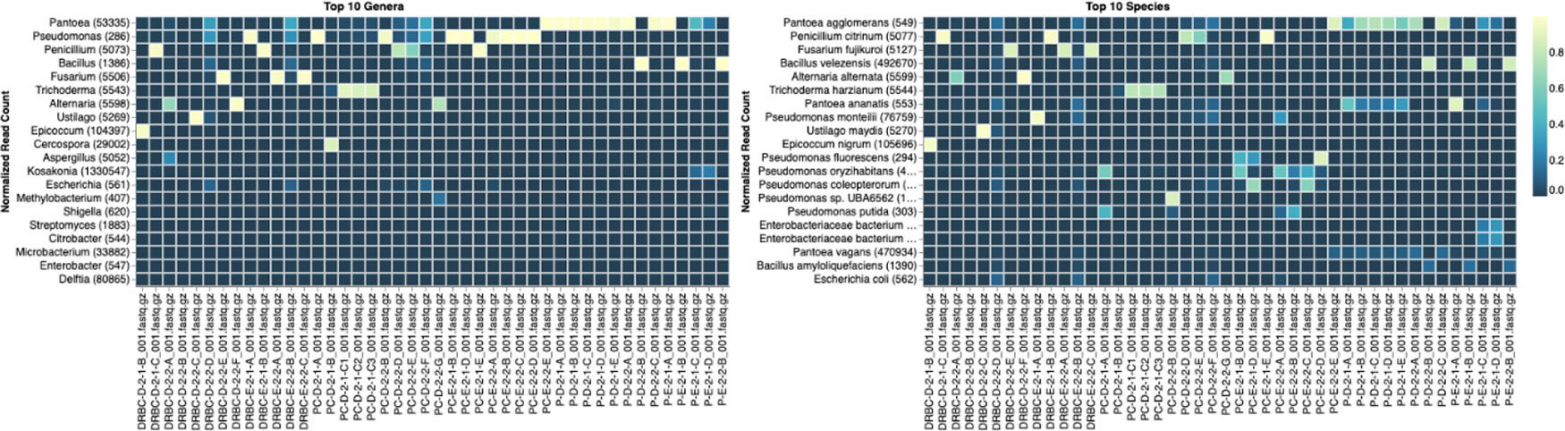
Summary heatmap of colony classification by whole genome sequencing.

Each colony which was imaged on plates and chosen for whole genome sequencing and OneCodex analysis is displayed in Figure 1 (DRBC), Figure 2 (PDA-chloramphenicol) and Figure 3 (PDA no chloramphenicol). A link to each OneCodex analysis and its respective NCBI submission ID is available in Supplementary Table 1 - Sheet Summary ( *Extended data*, McKernan *et al*., 2021). Some of the colonies from the plate merged with other colonies producing mixtures of genomes as evident in the OneCodex pie charts. These merged colonies were further evidenced by the display of bimodal sequence coverage (clusters of contigs at 1000X and 10X coverage) and compared with the plating images ( Figure 4). A heatmap of sequencing read speciation and purity is seen in Figure 5. While merged colonies can be difficult to resolve visually, whole genome sequencing can resolve simple metagenomes and still extract additional diversity information from the samples. Colonies that were noticeably mixed according to sequence analysis and colony visual inspection were more prevalent with the PDA without selection colonies ( Table 1).

**Table 1.** Summary of colony forming unit classification.

	Bacteria	Fungi	Mixed
DRBC CAMP	3	12	2
PDA CAMP	10	9	1
PDA No CAMP	14	4	4

**Figure 6.**
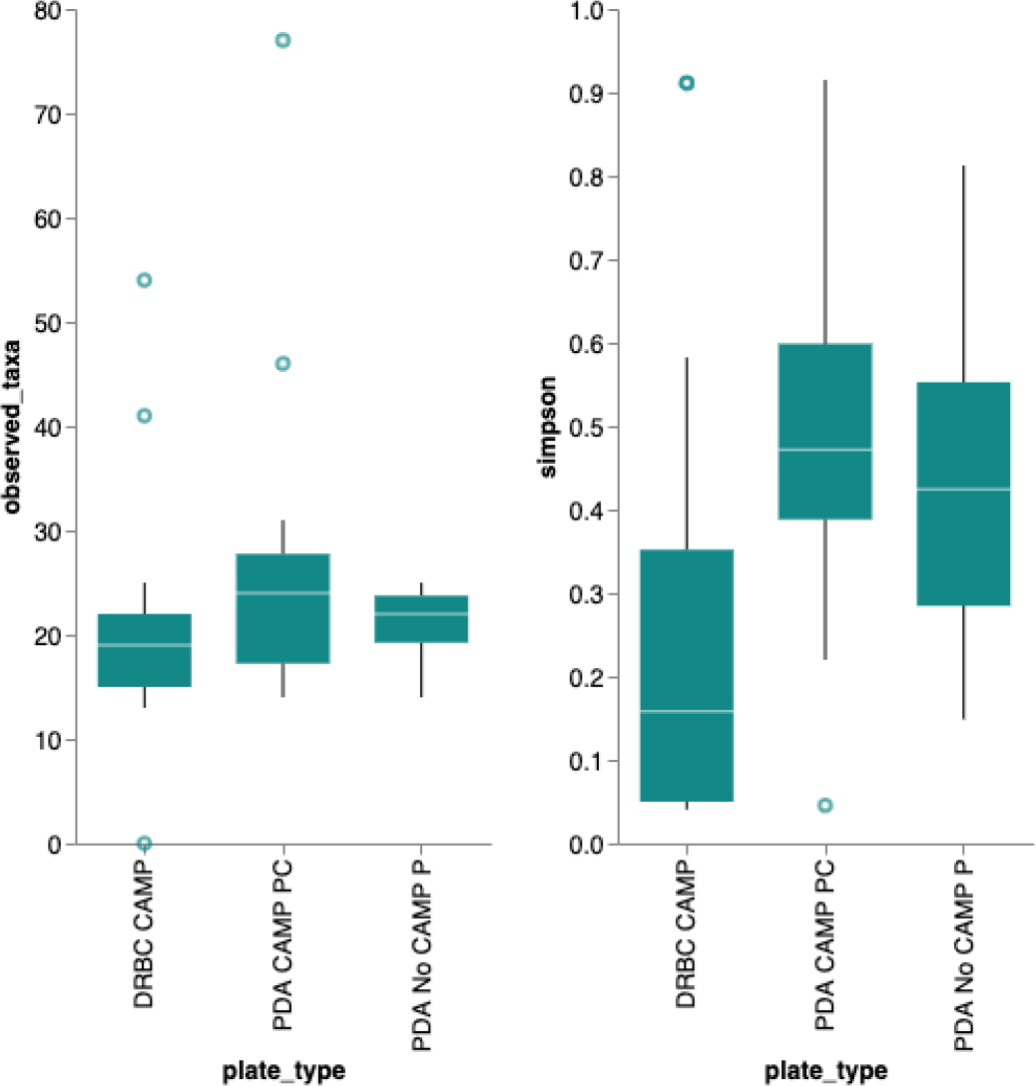
Simpson’s Diversity Index. Simpson’s diversity index ( https://geographyfieldwork.com/Simpson%27sDiversityIndex.htm) is used to quantify the biodiversity of a habitat on a 0 to 1 scale. It takes into account the number of species present, as well as the relative abundance of each species. A diversity index of 1 represent infinite diversity where 0 reflects no diversity. Dichloran Rose Bengal (DRBC) plating demonstrates the lowest diversity. This is not surprising given DRBC contains 3 different selection agents. While this limits bacterial contamination it also limits yeast and mold growth.

A Simpson’s diversity index analysis demonstrated PDA with CAMP provides the highest diversity score ( Figure 6) While the DRBC had 100-fold lower CFU counts than PDA without selection, it predominantly displayed fungal colonies (80%) while PDA without selection was biased toward bacteria (22%). PDA with chloramphenicol displayed more fungi (55%) than bacteria and also produced a half log more fungal colonies than DRBC with chloramphenicol ( Table 2).

**Table 2.** Cannabis samples plated on 3 different media. Plating on different media demonstrates a LOG scale difference in Colony Forming Units (CFU) with each plating medium. Sequencing can attribute only half of the colonies as bacteria on Potato Dextrose Agar (PDA) with chloramphenicol. This implies a 5-fold under counting of yeast and mold on Dichloran Rose Bengal (DRBC).

Sample	DRBC					
	10 ^-2^ CFU/g	10 ^-2^ CFU/g	10 ^-2^ CFU/g	10 ^-3^ CFU/g	10 ^-3^ CFU/g	10 ^-3^ CFU/g
Low A	0	4	1	1	0	0
Low B	0	3	1	1	1	0
Low C	2	1	0	0	0	0
Low D	2	5	2	0	2	0
Low E	2	0	2	0	0	0
			Average CFU/g			

Sample	PDA with Chloramphenicol					
	10 ^-2^ CFU/g	10 ^-2^ CFU/g	10 ^-2^ CFU/g	10 ^-3^ CFU/g	10 ^-3^ CFU/g	10 ^-3^ CFU/g
Low A	8	16	12	1	3	2
Low B	8	12	8	1	0	3
Low C	13	19	13	1	2	1
Low D	3	12	21	2	0	1
Low E	9	7	4	0	3	3
			Average CFU/g			

Sample	PDA with Chloramphenicol					
	10 ^-2^ CFU/g	10 ^-2^ CFU/g	10 ^-2^ CFU/g	10 ^-3^ CFU/g	10 ^-3^ CFU/g	10 ^-3^ CFU/g
Low A	127	133	124	32	32	21
Low B	151	157	101	26	20	28
Low C	TNTC	TNTC	TNC	41	45	37
Low D	147	141	123	32	26	26
Low E	138	102	119	23	15	24
			Average CFU/g			

One fungal sample ( *Cladosporum*) presented delayed Ct (31.79) with PathoSEEK Total Yeast and Mold (ITS3-TYM) qPCR primers. Scrutiny of the primer sequences against the *Cladosporum* genome shows proper primer binding locations but missing probe sequences. This genome has low coverage (10X) and the repetitive ITS qPCR target regions are often poorly assembled in low coverage-genomes. This may explain the missing probe sequence in the low coverage fragmented assembly. Additionally, some significantly delayed PathoSEEK Total Aerobic Count (TAC) signal was observed in fungal colonies. This is the result of the use of the lytic enzyme (TLP) which is cloned and expressed in *E. coli* and contains some background *E.coli* DNA. This background TLP expression in *E. coli* produces signals that can be seen in blank preparations. In some cases, this signal is elevated due to mixed colonies observed in the sequencing data.

The qPCR method represents an increased selectivity in assessing fungal and bacterial CFU compared to DRBC, where only -92% of the colonies were fungal colonies. Quantitative PCR identified all fungi and never mistook one for bacteria. In a minority of cases we had visually mixed colonies. Even if we discount the mixed colonies and count, only the single bacterial colony out of 13 on DRBC, we obtain 92% (1/13) fungal colonies on DRBC where qPCR delivered perfect results. As a comparison, quantitative PCR demonstrated over 10 Cts (1024 fold) differences between the TYM and TAC signals on fungal colonies. The majority of the residual TAC signal being observed in fungi can be normalized and discounted with the background *E. coli* TLP DNA signal measured in blank preparations.

**Figure 7.**
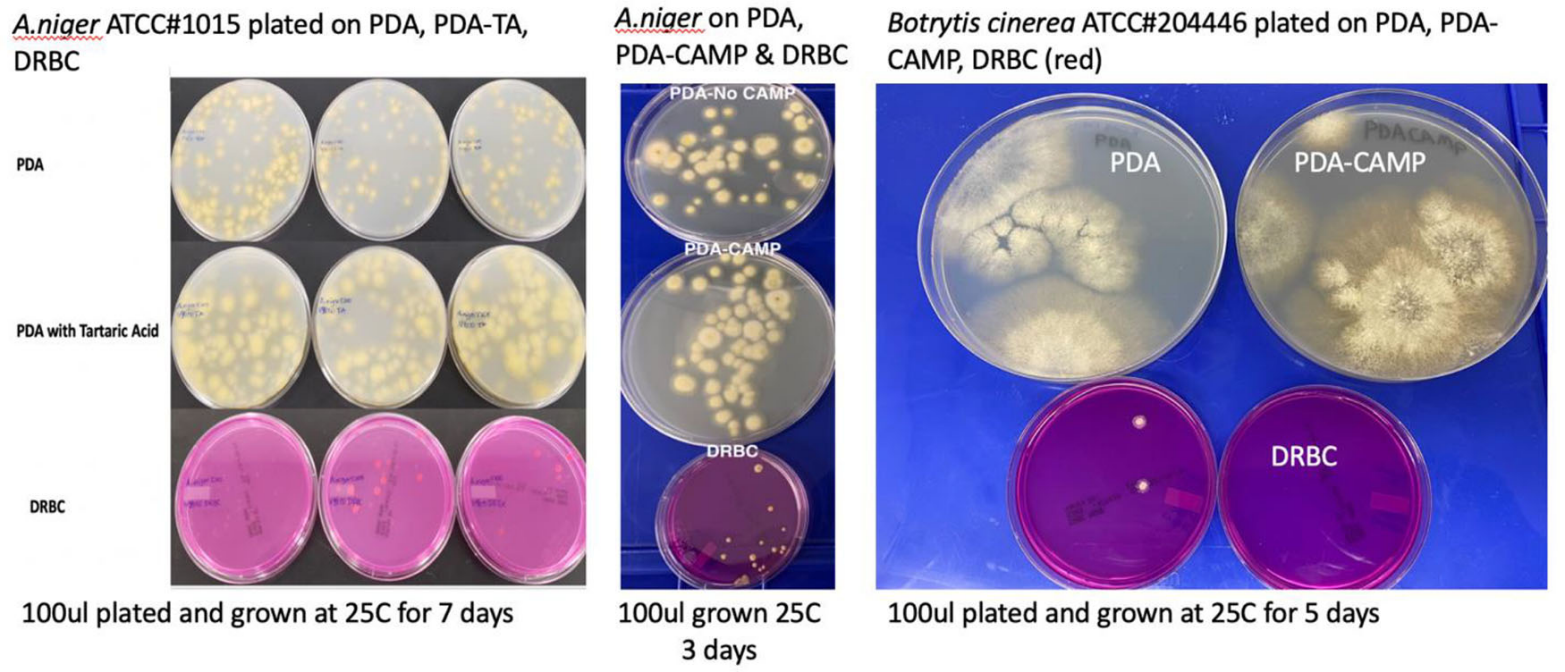
*Aspergillus niger* and *Botrytis cinerea* monocultures plated on 3 different medias. Cultures were plated on Potato Dextrose Agar (PDA), PDA with selection (chloramphenicol or Tartaric acid), and Dichloran Rose Bengal (DRBC). Fewer colonies are consistently found on DRBC.

To confirm these observations several *Aspergillus* species and *Botrytis cinerea* were ordered from ATCC and plated on various plating medias in absence of background cannabis matrix ( Table 3 and Figure 7). In all cases DRBC showed reduced CFU counts.

**Table 3.** Mono-culture evaluations. Fungi species were ordered from American Tissue Culture Collection (ATCC) and plated on 2 different medias (PDA and DRBC) to assess growth performance of the organisms in absence of cannabis background bacteria and matrix.

Species	ATCC Number	qPCR	DRBC Plating	PDA
*Aspergillus brasiliensis*	16404	Amp	5 Day Growth	5 Day Growth
*Aspergillus flavus*	9643	Amp	Less growth in 7 Days	5 Day Growth
*Aspergillus fumigatus*	204305	Amp	Less growth in 7 Days	5 Day Growth
*Aspergillus niger*	16888	Amp	Less growth in 7 Days	5 Day Growth
*Aspergillus terreus*	1012	Amp	Less growth in 7 Days	5 Day Growth
*Aspergillus tubigensis*	1004	Amp	Less growth in 7 Days	5 Day Growth
*Candida tropicalis*	13803	Amp	5 Day Growth	5 Day Growth
*Penicillium breviocompactum*	9056	Amp	Less growth than PDA	5 Day Growth
*Purpureocillium lilacinum*	10114	Partial Amp	5 Day Growth	5 Day Growth
*Rhizopus oryzae*	52748	Partial Amp	Less growth than PDA	5 Day Growth

## Discussion

Microbial media and their selection have a significant impact on the Simpson’s diversity index of microbes observed with whole genome sequencing. This has been noted in prior microbiome surveys in cannabis, in which culturing the microbes changes the representation of the microbiome as measured by qPCR and sequencing performed directly off of the flower ( McKernan *et al.*, 2015, 2016). In this study the DRBC selection reduced bacterial growth more than PDA with chloramphenicol, but also reduced the fungal CFU 5-fold in the process. This has important implications for chloramphenicol-sensitive cannabis endophytes like *Aspergillus*, *Pythium* and *Fusarium.* Both media types (PDA and DRBC) are referenced in the FDA Bacteriological Analytical Manual. States exclusively considering DRBC for ease of colony visualization should be aware of the species-specific sensitivities of using a single medium type, and consider species-specific testing for such pathogenic organisms, to complement a partial yeast and mold test offered from a single selection-based medium. PCR-based techniques can identify more organisms than DRBC alone as no selection is occurring given thorough cell lysis is achieved for qPCR analysis.

Plating also suffers from having a very limited dynamic range. Since it is difficult to count colonies when more than 100 colonies are present on a plate, multiple dilutions are often required to understand the full range of CFU counts one may encounter with a test which is attempting to quantify 10,000 CFUs/gram. This results in multiplying diluted CFUs 10, 100 and even a 1,000 fold to back-estimate the total CFU count. In this scenario a single colony can swing the CFU count from passing to failing (9 colonies x 1,000 fold dilution vs 10 colonies at 1,000 fold dilution). Quantitative PCR has a linear dynamic range over 5-6 orders of magnitude and no such multiplication is required. Thus, qPCR provides a more accurate itemization of actual CFUs counts.

*In-vitro* inclusion and exclusion testing with ITS3 qPCR on ATCC-sourced organisms demonstrated over 96% inclusion (50 yeast and mold) and zero bacterial cross reactivity (30 bacteria) ( *Extended data:* Supplementary Table 1- Sheet TYM Inclusion & TYM Exclusion). *In-silico* analysis of ITS3 primer sequences, predicts over 1400 yeast and mold should amplify with the described ITS3 primer sequences. All plating media, even with three different forms of selection (DRBC), had bacterial contamination and each level of selection reduced fungal CFU counts.

## Data availability

### Underlying data

NCBI Bioproject: Under Counting of Total Yeast and Mold on Cannabis using DRBC, Accession number PRJNA725256, https://www.ncbi.nlm.nih.gov/bioproject?term=PRJNA725256.

### Extended data

Zenodo: Whole genome sequencing of colonies derived from cannabis flowers and the impact of media selection on benchmarking total yeast and mold detection tools, https://doi.org/10.5281/zenodo.4759883 ( McKernan *et al*., 2021).

This project contains the following extended data:

Summary Table 1: OneCodex URLs and NCBI BioSample IDs for every sample.

TYM Inclusion: ATCC organisms tested for inclusion criteria

TYM Exclusion: ATCC organisms tested for exclusion criteria

Sequencing: Number of reads, Read Pairs and Total Gigabases sequenced for each sample.

Assembly: Complete Assembly statistics for each sample generated by QUAST

Data are available under the terms of the Creative Commons Attribution 4.0 International license (CC-BY 4.0).
