# Supplementary material for: Whole genome sequencing of colonies derived from cannabis flowers and the impact of media selection on benchmarking total yeast and mold detection tools
Source: F1000Res. 2021 Aug 12;10:624. Originally published 2021 Jul 21. [Version 2] doi: 10.12688/f1000research.53467.2 (PMC8383127; doi:10.12688/f1000research.53467.2)
Supplement: Peer review history

# Peer review: Reviewer response for version 2

**DOI:** 10.5256/f1000research.70512.r91814

**Published:** 2021-08-23

**Authors:** Zamir K. Punja

**Affiliations:** 1 Department of Biological Sciences, Simon Fraser University, Burnaby, BC, Canada

I approve the indexing of this revised paper with no reservations.

Is the rationale for developing the new method (or application) clearly explained?

Yes

Is the description of the method technically sound?

Yes

Are the conclusions about the method and its performance adequately supported by the findings presented in the article?

Yes

If any results are presented, are all the source data underlying the results available to ensure full reproducibility?

Yes

Are sufficient details provided to allow replication of the method development and its use by others?

Yes

Reviewer Expertise:

microbiology, plant pathology, plant biology, cannabis pathogens, post-harvest quality

I confirm that I have read this submission and believe that I have an appropriate level of expertise to confirm that it is of an acceptable scientific standard.

# Peer review: Reviewer response for version 1

**DOI:** 10.5256/f1000research.56852.r90093

**Published:** 2021-08-04

**Authors:** Zamir K. Punja

**Affiliations:** 1 Department of Biological Sciences, Simon Fraser University, Burnaby, BC, Canada

- Add “human pathogenic” where pathogenic is mentioned as the concern is with these particular microbes and not those potentially that could be affecting the plant as plant pathogens.
- The inclusion of the 3 selection media should be elaborated on. Why were these particular 3 media selected? Provide references to show where, or in what capacity, they may have been used in previously published work. There is abundant published work on the use of PDA with antibiotics to isolate fungi in the plant pathology literature. In fact, it is a standard medium used for isolation in labs worldwide. The addition of dichloran and rose bengal have also been used to restrict the growth of certain groups of fungi and bacteria as a semi-selective medium for isolation in particular from soil samples. Therefore, it would not be expected to provide a broad spectrum of recovery of fungi and yeasts. It is surprising that this medium would be used to assess total yeast and mold counts in cannabis.
- The comparison of the 3 media in this study sheds light on the differences in levels of recovery of fungi and yeasts. This is an important finding – not all media behave in the same manner. To observe a 10-fold difference in recovery between these media is quite significant as it illustrates the potential for under-representation in the recovery process.
- The use of whole genome sequencing to apply to the identification of colony-forming units is a definite plus for this work. It shows the ability to rapidly identify what is present on the culture media with regard to molds that originated from the samples.
- There are several prior reports of authors having recovered a range of fungi from cannabis buds and identified them using the ITS region. Please include these as a reference by which to compare the fungi and yeasts identified in the present study. It is important to build a body of knowledge on the exact identity of the general and species found on cannabis and how prevalent they are.
- The report of endophytes in cannabis should be accompanied by a reference citation. These particular microbes are more difficult to recover in culture media and therefore a molecular approach has merit.
- The inclusion of confirmed ATCC culture specimens to demonstrate differences in growth on the 3 media is a good confirmatory experiment.
- The cannabis samples that originated from Steadfast Analytical Laboratories would have had an analysis of total yeast and mold conducted on them. Is it possible to have these results compared to those from the present study to show how the commercial lab testing may differ from the current study? Or was that not an objective of the current study?
- During the preparation of samples for the ITS3 qPCR procedure, was there a subset of samples included that did not contain the TLP lysis step to show that it made a difference? Or is that included in prior published work?
- For the 45 colonies that were selected for whole genome sequencing, could the identified genus and species be presented in a separate table? Perhaps in accordance with the media from which they were derived from? These would be a summary of what is shown in Figures 1, 2, 3. This is in addition to the OneCodex analysis and the NCBI submission ID available in Supplementary Table 1 It also helps clarify the data shown in Figure 5.
- The Simpson’s diversity index analysis shown in Figure 6 is extremely helpful to show the differences between the 3 media types in recovery.
- The results from qPCR of the homogenate that was collected from the Whirl-Pak bags and subjected to PathoSEEK. How did this compare with the colony identification of the same sample plated on the 3 different media with regards to the identification of the genus and species present? Can this be shown in a Table?
- The use of DRBC, if conducted by testing laboratories, is worrisome. It is known that the addition of dichloran and rose bengal is specifically used to discourage certain types of microbes from growing when used for recovery of specific types of fungi from soil samples. The inclusion of DRBC in a testing laboratory for cannabis TYM counts should be discouraged, as shown in the present work where total CFU’s recovered were significantly lower compared to PDA with chloramphenicol. DRBC would significantly under-estimate the TYM counts as shown in Figure 4.
- In Table 2, the headings seem incorrect as there are two with “PDA with chloramphenicol” and one should be “PDA w/o chloramphenicol”
- Table 3 and Figure 7 clearly show how DRBC provides reduced growth compared to PDA.

Overall, this is an informative study and the results merit publication. Once the items identified by the reviewer are addressed, this study will be a good addition to the slowly expanding studies showing how complex the assessment of total yeast and mold levels in cannabis is. The information from these types of studies should guide government agencies on the pitfalls of certain methods used to assess TYMC.

Is the rationale for developing the new method (or application) clearly explained?

Yes

Is the description of the method technically sound?

Yes

Are the conclusions about the method and its performance adequately supported by the findings presented in the article?

Yes

If any results are presented, are all the source data underlying the results available to ensure full reproducibility?

Yes

Are sufficient details provided to allow replication of the method development and its use by others?

Yes

Reviewer Expertise:

microbiology, plant pathology, plant biology, cannabis pathogens, post-harvest quality

I confirm that I have read this submission and believe that I have an appropriate level of expertise to confirm that it is of an acceptable scientific standard, however I have significant reservations, as outlined above.

# Response

**Published:** 2021-08-05

**Authors:** Kevin McKernan

**Affiliations:** Medicinal Genomics Corporation, USA

Thank you for this valuable feedback. I have interweaved our responses below and will update the manuscript accordingly:

- *Add “human pathogenic” where pathogenic is mentioned as the concern is with these particular microbes and not those potentially that could be affecting the plant as plant pathogens.*
- **Response: **Agree. Done
- *The inclusion of the 3 selection media should be elaborated on. Why were these particular 3 media selected? Provide references to show where, or in what capacity, they may have been used in previously published work. There is abundant published work on the use of PDA with antibiotics to isolate fungi in the plant pathology literature. In fact, it is a standard medium used for isolation in labs worldwide. The addition of dichloran and rose bengal have also been used to restrict the growth of certain groups of fungi and bacteria as a semi-selective medium for isolation in particular from soil samples. Therefore, it would not be expected to provide a broad spectrum of recovery of fungi and yeasts. It is surprising that this medium would be used to assess total yeast and mold counts in cannabis.*
- **Response:** We were not involved in the selection of these media types. The study was initiated on PDA in Michigan. After we completed the PDA study, the organizers informed us they were switching to DRBC based on Steadfast having used chloramphenicol based culture platforms to categorize the samples before shipping them to other labs in Michigan. We suspect the 3 media types were chosen due to their presence in the FDA BAM. We have added a sentence to clarify this.
- *The comparison of the 3 media in this study sheds light on the differences in levels of recovery of fungi and yeasts. This is an important finding – not all media behave in the same manner. To observe a 10-fold difference in recovery between these media is quite significant as it illustrates the potential for under-representation in the recovery process. The use of whole genome sequencing to apply to the identification of colony-forming units is a definite plus for this work. It shows the ability to rapidly identify what is present on the culture media with regard to molds that originated from the samples. There are several prior reports of authors having recovered a range of fungi from cannabis buds and identified them using the ITS region. Please include these as a reference by which to compare the fungi and yeasts identified in the present study. It is important to build a body of knowledge on the exact identity of the general and species found on cannabis and how prevalent they are.*
- **Response: **Very good point. We have added a paragraph to describe the substantial prior art here.
- *The report of endophytes in cannabis should be accompanied by a reference citation. These particular microbes are more difficult to recover in culture media and therefore a molecular approach has merit.*
- **Response: **Very good point. We have added references to emphasize Dichlorans inhibitory nature.
- *The inclusion of confirmed ATCC culture specimens to demonstrate differences in growth on the 3 media is a good confirmatory experiment.*
- **Response: **This is an important control but it should be known by people in the field that AOAC certification doesn’t require the inclusion or exclusion testing to be performed in the presence of matrix and this inclusion and exclusion criteria can be obtained using a different media than DRBC. We believe this provides a false sense of safety. This is challenging to perform in the presence of matrix as there is no supplier of sterilized cannabis that ship across state lines and inclusion and exclusion testing can be impacted by background microbial content. Further legalization will improve this.
- *The cannabis samples that originated from Steadfast Analytical Laboratories would have had an analysis of total yeast and mold conducted on them. Is it possible to have these results compared to those from the present study to show how the commercial lab testing may differ from the current study? Or was that not an objective of the current study?   *     
- **Response:** As part of the AOAC study, we were blinded from these data. We do know that the high, medium and low categories were allocated according to culture-based methods that used CAMP and that quantitative PCR over estimated microbial burden on the low DRBC samples.
- *During the preparation of samples for the ITS3 qPCR procedure, was there a subset of samples included that did not contain the TLP lysis step to show that it made a difference? Or is that included in prior published work?*
- **Response: **We did not include TLP lysis versus no TLP as the study required we settle on a single method for evaluation. We are in the process of writing up that comparison for another publication.
- *For the 45 colonies that were selected for whole genome sequencing, could the identified genus and species be presented in a separate table? Perhaps in accordance with the media from which they were derived from? These would be a summary of what is shown in Figures 1, 2, 3. This is in addition to the OneCodex analysis and the NCBI submission ID available in Supplementary Table 1 It also helps clarify the data shown in Figure 5.*
- **Response: **This is an important point. This does exist in Figure 5 but we failed to clarify the sample nomenclature that clarifies this. We have added a sample key to describe which samples are DRBC, PDA-CAMP, PDA-no-CAMP.
- *The Simpson’s diversity index analysis shown in Figure 6 is extremely helpful to show the differences between the 3 media types in recovery.The results from qPCR of the homogenate that was collected from the Whirl-Pak bags and subjected to PathoSEEK. How did this compare with the colony identification of the same sample plated on the 3 different media with regards to the identification of the genus and species present? Can this be shown in a Table? The use of DRBC, if conducted by testing laboratories, is worrisome. It is known that the addition of dichloran and rose bengal is specifically used to discourage certain types of microbes from growing when used for recovery of specific types of fungi from soil samples. The inclusion of DRBC in a testing laboratory for cannabis TYM counts should be discouraged, as shown in the present work where total CFU’s recovered were significantly lower compared to PDA with chloramphenicol. DRBC would significantly under-estimate the TYM counts as shown in Figure 4.*
- **Response:** We were only allowed to ship cultures on plates across state lines. As a result, we have Cq scores for the colonies that were picked and isolated in Figures 1,2,3 under the TYM and TAC Cq columns on the right. This only informs on inclusion and exclusion capabilities of the primers for the colonies harvested but loses quantitative information. We were not allowed to ship homogenized matrix in the mail to assess the Cq prior to plating. Labs local to Michigan have performed this comparison and are free to publish those results. The summary of the results communicated to us were that the qPCR had better concordance with PDA with CAMP and over estimated CFUs on the DRBC Low samples. This significantly differs from Michigans stated intentions with the ERV where they voiced concerns about molecular methods undercounting risk (https://help.medicinalgenomics.com/hubfs/Regulatory%20Info%20for%20Sales/Michigan%20MRA%20Notification%20of%20additional%20validation%20requirements%20August%202020.pdf). The opposite turned out to be true. DRBC is undercounting risk compared to qPCR. The MRA was also led to believe that Klebsiella was not an appropriate validation organism as it was not commonly found on cannabis despite it having been published by Thompson et al previously. Candida albicans (which we have never seen documented on Cannabis) was prioritized as a CRM. We have added some language to address this to the best of our ability.
- *In Table 2, the headings seem incorrect as there are two with “PDA with chloramphenicol” and one should be “PDA w/o chloramphenicol”Table 3 and Figure 7 clearly show how DRBC provides reduced growth compared to PDA.*
- **Response: **Thank you!. Good catch. We have updated that.

# Peer review: Reviewer response for version 1

**DOI:** 10.5256/f1000research.56852.r90094

**Published:** 2021-07-28

**Authors:** Cindy Orser

**Affiliations:** 1 CLIP Laboratories, San Diego, CA, USA

A well-executed study of considerable breath and size to further solidify the argument against State regulatory bodies requiring agar plating to evaluate microbial load on cannabis flower in lieu of proven superiority of molecular assays. In addition, this study demonstrates confirmation of chloramphenicol's ability to knock down *Fusarium *and *Aspergillus* growth when plating on DRBC at relatively low chloramphenicol levels [~0.1 mg/mL], not sure of the concentration in PDA agar. Nonetheless, this study should finally put to rest the issue of which method is most accurate at representing microbial load on cured cannabis flower. With qPCR assays shown to be fundamentally superior to culturing and plating, the discussion should now move over to "sampling" and how inadequate the normal sample size per batch size is to give a glimpse into the microbiome of the cannabis flower and the intrigue of how cultivation methods influence the cannabis microbiome.

Is the rationale for developing the new method (or application) clearly explained?

Yes

Is the description of the method technically sound?

Yes

Are the conclusions about the method and its performance adequately supported by the findings presented in the article?

Yes

If any results are presented, are all the source data underlying the results available to ensure full reproducibility?

Yes

Are sufficient details provided to allow replication of the method development and its use by others?

Yes

Reviewer Expertise:

plant microbiology; cannabis analytical testing; diagnostics

I confirm that I have read this submission and believe that I have an appropriate level of expertise to confirm that it is of an acceptable scientific standard.

# Response

**Published:** 2021-08-10

**Authors:** Kevin McKernan

**Affiliations:** Medicinal Genomics Corporation, USA

Thank you for this feedback. The sampling comment is very pertinent. We were not in control of this aspect of the study so opted to save this topic for another manuscript. AOAC communicated to us that many different cannabis samples were mixed to normalize chemotype affects on culture. This is a very good idea as we have seen chemotype specific effects on cannabis microbiology and its published to occur in *Trema orientalis* (https://pubmed.ncbi.nlm.nih.gov/34035994/).

 One of the concerns with plating, is that the antibiotic cannabinoids and terpenes may get liberated from trichomes in the aggressive lab homogenization and media saturation process. This may influence the viability of some of the microbes. This aggressive homogenization and fluid saturation is not what a consumer experiences. 

 Many publications demonstrate the antibiotic nature of cannabinoids and how different cannabinoids exhibit different antibiotic properties thus we should expect different chemotypes to plate differently given there is no purification step prior to plating (https://www.ncbi.nlm.nih.gov/pmc/articles/PMC7105690/). Molecular methods lyse open cells and purify the DNA away from such potential growth inhibitors with Ethanol extractions. 

 To confirm these samples were indeed a mixture of different cannabis samples, we were sent DNA from these mixtures and performed 10Mb SureSelect capture and deep Illumina Sequencing on these samples to under stand how well they were mixed and how diverse they where. The read genotypes indeed suggested more than a single cannabis sample was present and likely more than 4 in each High, Medium and Low Categories. 

 We put these data public for anyone who is interested but felt it would bloat this manuscript with confirmatory data and distract from the core focus of the study. 

https://www.kannapedia.net/strains/rsp11748/

https://www.kannapedia.net/strains/rsp11749/

https://www.kannapedia.net/strains/rsp11750/

https://www.kannapedia.net/strains/rsp11752/

https://www.kannapedia.net/strains/rsp11753/

https://www.kannapedia.net/strains/rsp11754/

https://www.kannapedia.net/strains/rsp11755/

https://www.kannapedia.net/strains/rsp11756/

https://www.kannapedia.net/strains/rsp11757/

https://www.kannapedia.net/strains/rsp11758/

https://www.kannapedia.net/strains/rsp11759/

https://www.kannapedia.net/strains/rsp11760/

https://www.kannapedia.net/strains/rsp11761/

https://www.kannapedia.net/strains/rsp11762/
